# Ethical theories, governance models, and strategic frameworks for responsible AI adoption and organizational success

**DOI:** 10.3389/frai.2025.1619029

**Published:** 2025-07-16

**Authors:** Mitra Madanchian, Hamed Taherdoost

**Affiliations:** ^1^Department of Arts, Communications, and Social Sciences, School of Arts, Science, and Technology, University Canada West, Vancouver, BC, Canada; ^2^GUS Institute, Global University Systems, London, United Kingdom

**Keywords:** ethical frameworks, algorithmic fairness, stakeholder responsibility, risk assessment, governance models, organizational culture

## Abstract

As artificial intelligence (AI) becomes integral to organizational transformation, ethical adoption has emerged as a strategic concern. This paper reviews ethical theories, governance models, and implementation strategies that enable responsible AI integration in business contexts. It explores how ethical theories such as utilitarianism, deontology, and virtue ethics inform practical models for AI deployment. Furthermore, the paper investigates governance structures and stakeholder roles in shaping accountability and transparency, and examines frameworks that guide strategic risk assessment and decision-making. Emphasizing real-world applicability, the study offers an integrated approach that aligns ethics with performance outcomes, contributing to organizational success. This synthesis aims to support firms in embedding responsible AI principles into innovation strategies that balance compliance, trust, and value creation.

## 1 Introduction

Artificial intelligence (AI) is transforming technology and science, reshaping human-technology interactions, problem-solving, and our understanding of intelligence (Schwaeke et al., 2024; Glikson and Woolley, 2020; Chalutz-Ben, 2023; Alzubi et al., 2025). As a core driver of the Fourth Industrial Revolution, AI’s growing adoption is reshaping organizational and business processes (Cubric, 2020; Loureiro et al., 2021; Ransbotham et al., 2017; Kvitka et al., 2024). For example, research analyzing 42 countries found a 10% increase in AI intensity (measured by AI patents per capita) correlates with a 0.3% increase in GDP, with stronger effects in high-income countries and service sectors (Gondauri, 2025). Despite its promise to boost global GDP and drive innovation (Jan et al., 2020; Arsenijevic and Jovic, 2019; Verma et al., 2021), AI implementation remains challenging, with a high failure rate (Ransbotham et al., 2017; Bitkina et al., 2020; Duan et al., 2019). Studies show that approximately 70% of companies report minimal impact from AI, and only about 13% of data science projects reach production (Ångström et al., 2023).

The rapid development and deployment of AI raise significant concerns about its societal impact, particularly relating to income inequality, human rights, and ethical considerations (McElheran et al., 2024; Hallamaa and Kalliokoski, 2022; Mantelero and Esposito, 2021; Taherdoost et al., 2025). Although substantial research has explored these issues from humanities, philosophy, and computer science perspectives (Kazim and Koshiyama, 2021), public engagement remains limited despite the human-centric imperative of ethical AI (Kieslich et al., 2022). AI technologies should be developed and used in ways that respect fundamental rights and adhere to ethical principles such as explainability, justice, autonomy, non-maleficence, and beneficence (Hauer, 2022; Galiana et al., 2024). As AI increasingly influences decision-making traditionally reserved for humans, its integration into scientific research also necessitates adherence to established ethical norms (Resnik and Elliott, 2019; Pennock, 2019; Resnik, 1996; Resnik and Hosseini, 2024). However, divergent views on AI’s definition and capabilities persist, and unforeseen consequences often arise despite good intentions (Munn, 2023; Anagnostou et al., 2022; Ashok et al., 2022; Coeckelbergh, 2020).

There are many different and substantial research gaps in the area of ethics surrounding the use of AI. One significant gap is the lack of focus on ethical implications in a number of fields, including marketing, finance, engineering, architecture, and construction, where problems with algorithmic bias, data privacy, and job loss are still little studied (Liang et al., 2024; Owolabi et al., 2024). A comprehensive ethical framework that can oversee the use of AI while addressing issues of accountability, transparency, and public trust is desperately needed (Owolabi et al., 2024; Azad and Kumar, 2024). A crucial translational gap that needs to be closed is highlighted by the discrepancy between the ethical AI concepts developed in academic literature and their actual use in industrial settings (Borg, 2022). Effective governance structures and best practices for ethical AI integration cannot be developed without interdisciplinary collaboration and continuous stakeholder communication (Azad and Kumar, 2024; Borg, 2022).

The purpose of this overview of the literature is to critically analyze how ethical issues are incorporated into the use of AI in a variety of industries. It looks for and evaluates current theories and models that support moral decision-making and governance in the application of AI. In the end, this analysis will offer insights for companies looking to strike a balance between innovation and ethical responsibility by highlighting the difficulties and best practices related to integrating ethics into AI systems through an examination of a wide spectrum of literature. With an emphasis on their applicability to contemporary AI activities and the consequences for stakeholders engaged in AI development and deployment, the scope includes theoretical frameworks, realistic models, and implementation methods.

The organization of this paper aims to offer a thorough analysis of the ethical integration of AI adoption. To guide responsible and performance-oriented AI adoption, this paper aims to examine ethical theories, particularly utilitarianism, deontology, and virtue ethics, and assess their relevance to AI-related decision-making in organizational contexts. It also explores governance models and stakeholder responsibilities that ensure fairness, transparency, and accountability in the design and deployment of AI systems. Further, the paper evaluates practical frameworks for risk assessment, explainability, and strategic alignment throughout the AI lifecycle. Finally, it proposes integrated strategies that translate ethical principles into actionable organizational practices, thereby fostering responsible AI adoption that contributes meaningfully to organizational success.

## 2 Theories of responsible AI adoption

Many studies in the field of AI ethics have been theoretical and conceptual in character (Seah and Findlay, 2021). The fact that there are so many AI ethics rules makes it difficult for practitioners to choose which ones to abide by. It should come as no surprise that research has been done to examine the constantly expanding list of particular AI principles (Siau and Wang, 2020; Mark and Anya, 2019). For instance, Jobin et al. (2019) examined 84 responsible AI standards and principles before coming to the conclusion that just five of them, transparency, fairness, non-maleficence, responsibility, and privacy, are primarily addressed and adhered to. Hagendorff (2020) conducted an analysis and comparison of 22 AI ethical principles to investigate their applicability in the fields of AI research, development, and application. The rising competitiveness among organizations to build strong AI tools has intensified the demand to establish ethical norms in AI (Vainio-Pekka, 2020).

These technologies are becoming more and more like active actors in our life, capable of influencing or even making decisions that were previously only made by humans. Due to this progression, in an increasingly automated and digitalized world, we must reevaluate and rethink what it means to be responsible, private, autonomous, and just. AI and ethics are closely related in both directions, and new ethical problems are raised by AI applications and capabilities (Galiana et al., 2024; Taherdoost, 2025).

### 2.1 Ethical theories and frameworks

The new ethical theories that take the distributed agency into account can help advance AI ethics. Conventional moral frameworks address individuals, and human responsibility assigns rewards or punishments according to personal choices and intentions. However, dispersed agency suggests that all players share accountability, which is the case with AI and, for instance, with firms, customers, software/hardware, designers, and developers (Taddeo and Floridi, 2018).

The most well-known examples are the many utilitarian perspectives that date back to Bentham (1789) and Mill (1861). They are predicated on the notion that, in theory, it is possible to total the benefits and drawbacks of a specific course of action. The ethically best choice is the one that has the maximum net utility, or utility less disutility. Theories of utilitarianism, often known as “consequentialist” or “teleological,” describe moral behavior that aims to maximize the “good” for the majority (Starr, 1983; McGee, 2010; Gustafson, 2013).

On the other hand, deontology is predicated on the idea that an action’s ethical assessment begins with the agent’s obligation while carrying it out. “Act only on that maxim by which you may at the same time will that it should become a universal law” is the most frequently cited expression of the categorical imperative (translation, given in Bowie, 2017). This categorical imperative prevents actors from justifying their own exemptions. Deontological theories, which have given rise to business social responsibilities (Mazutis, 2014; Lin et al., 2022), aim to explain moral behaviors as a set of standards or codes of conduct. Deontological ethics is still criticized today, usually for being overly strict and for potential contradictions between obligations. For instance, conflicting moral commitments might create situations in which following the rules to the letter may have unfavorable effects (O'Neill, 1989).

According to virtue theories, people behave in accordance with their inner “moral compass” when taking activities (White et al., 2023; James et al., 2023; Grant and McGhee, 2022). Some academics contend that deontology does not provide a workable framework for addressing moral disputes in everyday situations (Darwall, 2009). Table 1 summarizes key features of each ethical theory.

**Table 1:** Comparative overview of ethical theories applied to AI contexts.

Feature	Utilitarianism	Deontology	Virtue ethics	References
Focus	Consequences of actions	Duties and rules	Character of the moral agent	Bentham (1789)
Key Principle	Greatest happiness principle	Categorical imperative	Cultivation of virtues	O'Neill (1989)
Notable Philosophers	Jeremy Bentham, John Stuart Mill	Immanuel Kant	Aristotle, Elizabeth Anscombe	Hursthouse et al. (2018)
Strengths	Practical in policy-making	Clear moral guidelines	Emphasizes moral development	Buchanan et al. (2000)
Weaknesses	Can justify harmful actions	Rigid; may lead to moral dilemmas	Challenges in universal application	Berg (2020)

### 2.2 Responsible AI governance models

The term “responsible AI governance” refers to the systems put in place by businesses to deal with the moral questions raised by AI. Some of the most important ideas in responsible AI governance that have been discussed in the literature are stakeholder involvement, openness, justice, and accountability. According to Zhang and Lu (2021), these topics are essential for resolving public worries over prejudice, discrimination, and the possible abuse of AI systems.

Integrating responsible AI concepts into governance frameworks is crucial, according to a systematic review by Batool et al. (2023). To reduce the dangers of AI deployment and make sure that ethics are part of AI development from the start, this integration is crucial. There is a notable void in academic research and practical implementation, as the review finds no comprehensive frameworks that fully address these ideas.

Finding out who is responsible for AI system decisions is a major issue in AI governance. Organizational executives and legislators should also be involved in holding developers accountable (Morley et al., 2023). In order to gain the public’s trust, AI systems must be transparent. It is important for stakeholders to comprehend the decision-making process of AI systems. Users can understand the reasoning behind automated judgments with the help of explainable AI tools (Camilleri, 2024). Equal treatment of AI results is another important issue. A number of studies have brought attention to the possibility that algorithmic bias could cause discrimination against vulnerable populations (Batool et al., 2023). It is crucial to involve varied stakeholders, including affected communities, ethicists, and legal experts, when building frameworks for inclusive governance. The values and aspirations of society toward AI technology can be better understood with the help of stakeholder involvement (Mäntymäki et al., 2022).

The concept of corporate governance has evolved significantly over the years, leading to the development of various models that reflect different cultural, economic, and legal contexts. The most prominent models include the Anglo-American model, the Continental European model, and the Asian model. Each of these models presents unique features that influence corporate behavior and stakeholder relationships.

1. Anglo-American Model: Predominantly found in the United States and the United Kingdom, this model emphasizes shareholder primacy. It advocates for a board structure characterized by a unitary board system where executive and non-executive directors coexist. This model prioritizes transparency and accountability to shareholders, often leading to robust performance metrics (Maassen, 1999). However, critics argue that this focus on shareholder value can undermine other stakeholder interests, leading to short-termism in corporate strategies (Jensen, 2001).
2. Continental European Model: Common in countries like Germany and France, this model features a dual-board system comprising a management board and a supervisory board. The supervisory board oversees management activities while ensuring that stakeholder interests are represented (Mueller, 2006). This structure allows for greater oversight but can lead to slower decision-making processes due to its complexity. Proponents argue that it fosters long-term stability by balancing various stakeholder interests (Cheung et al., 2011).
3. Asian Model: This model varies widely across countries but often includes significant family ownership and control. In many Asian firms, family members dominate board positions, which can enhance decision-making efficiency but may also lead to conflicts of interest (Alalade et al., 2014). The Asian model emphasizes relationships and networks over formal governance structures, resulting in unique challenges regarding transparency and accountability.

A comparison shows how different governance models handle agency theory, conflicts between shareholders (principals) and management (agents). Performance-based compensation matches managerial incentives with shareholder interests under the Anglo-American paradigm (Grove et al., 2011), but it may overlook stakeholder concerns. The Continental European model’s dual-board structure provides additional scrutiny but may slow decision-making (Vig and Datta, 2018).

In these frameworks, emerging studies emphasize corporate social responsibility (CSR). Kaur and Singh (2018) discovered that organizations with excellent governance frameworks are more likely to engage in CSR, improving their long-term performance. Technology transforms corporate governance, according to recent research. Digital tools improve data reporting and stakeholder communication, increasing transparency (Adams et al., 2010).

The most often stated ethical principles in AI ethics standards are transparency, privacy, accountability, and fairness, according to a thorough literature assessment by Khan et al. (2022). These tenets form the cornerstone of legislative frameworks aimed at efficiently regulating AI technologies. A complete regulatory approach is demonstrated by the AI Act proposed by the European Commission, which creates a legislative framework that classifies AI systems according to risk categories. Before being introduced into the market, high-risk AI systems have to adhere to stringent safety, transparency, and accountability regulations (Kargl et al., 2022).

The High-Level Expert Group on AI created the Ethics Guidelines for Responsible AI in 2019, which outline seven more essential criteria that AI systems must fulfill: accountability, diversity, non-discrimination and fairness, privacy and data governance, human agency and oversight, technological robustness and safety, and transparency (Palumbo et al., 2024). The purpose of these guidelines is to establish a common norm for moral AI practices among EU member states.

### 2.3 Stakeholder theory and responsibility

AI adoption involves ethical, regulatory, and social responsibility. As AI is implemented, companies must manage significant ethical issues. AI deployment decisions require ethical frameworks, according to research. Binns (2018) suggests that AI system implementation should be transparent, fair, and accountable. A thorough literature study by Rjab et al. (2023) found that smart cities need ethical rules to mitigate AI technology dangers.

Organizational culture also influences responsible AI adoption. Shahzadi et al. (2024) found that ethical companies prioritize responsible AI activities. This includes encouraging employees to raise ethical and bias issues regarding AI systems. Organizational culture and accountability affect how AI technologies are perceived and used across sectors. According to the literature, organizations should actively work with lawmakers to create AI-specific rules. Organizations can encourage innovation and public interest by participating in regulatory framework discussions (Madan and Ashok, 2023).

Responsible AI requires stakeholder participation. Sharma (2024) advises stakeholders to address AI’s ethical effects on human interactions and society. The author claims that AI systems change human interactions and ethical norms as they become more incorporated into daily life. AI in transportation or healthcare can revolutionize how people interact with each other and technology. This requires parties to work together to prevent AI from undermining human values and social cohesion.

AI transparency and accountability needs vary by stakeholder. Hind et al. (2019) found that regulators want AI systems to be fair and safe, whereas end-users want explanations to develop trust and improve decision-making. This divergence emphasizes the need for stakeholder-specific communication techniques. Weller (2019) also notes that developers need system performance data to debug and improve, demonstrating the interdependence of stakeholder roles in responsible AI implementation.

**Figure 1:**
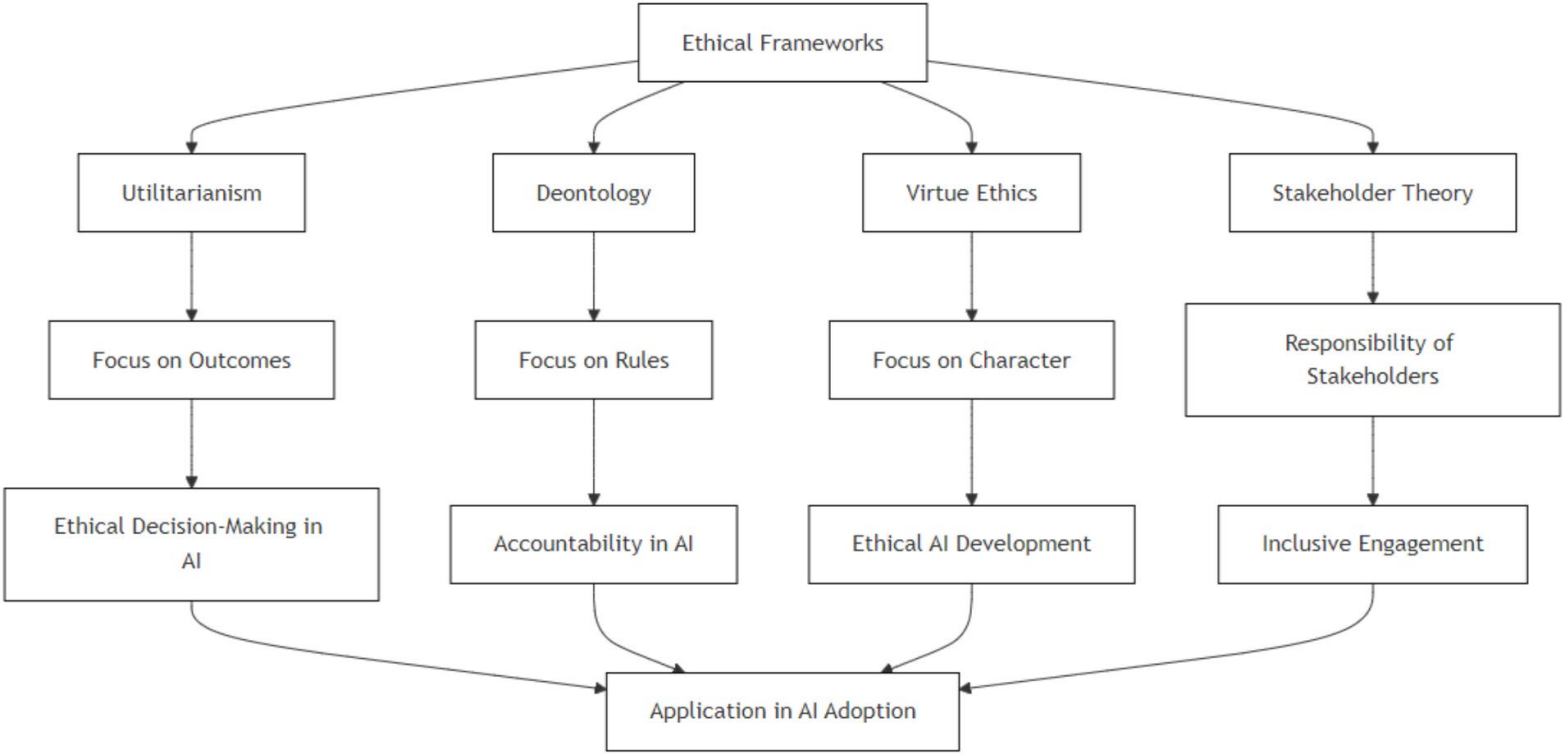
Comparative analysis of ethical frameworks.

For stakeholder decisions, responsible AI frameworks are needed. Deshpande and Sharp (2022) list individuals, organizations, and international bodies as responsible AI system stakeholders. Their research implies that collaborative ethical rules can improve AI responsibility at all levels. Stakeholder viewpoints in AI governance can reduce technological misuse concerns and benefit society. Figure 1 focuses on ethical frameworks but does not encompass broader organizational enablers like culture or leadership. These factors are addressed separately in Section 4 as part of the implementation strategy layer.

## 3 Models for responsible AI integration

**Figure 2:**
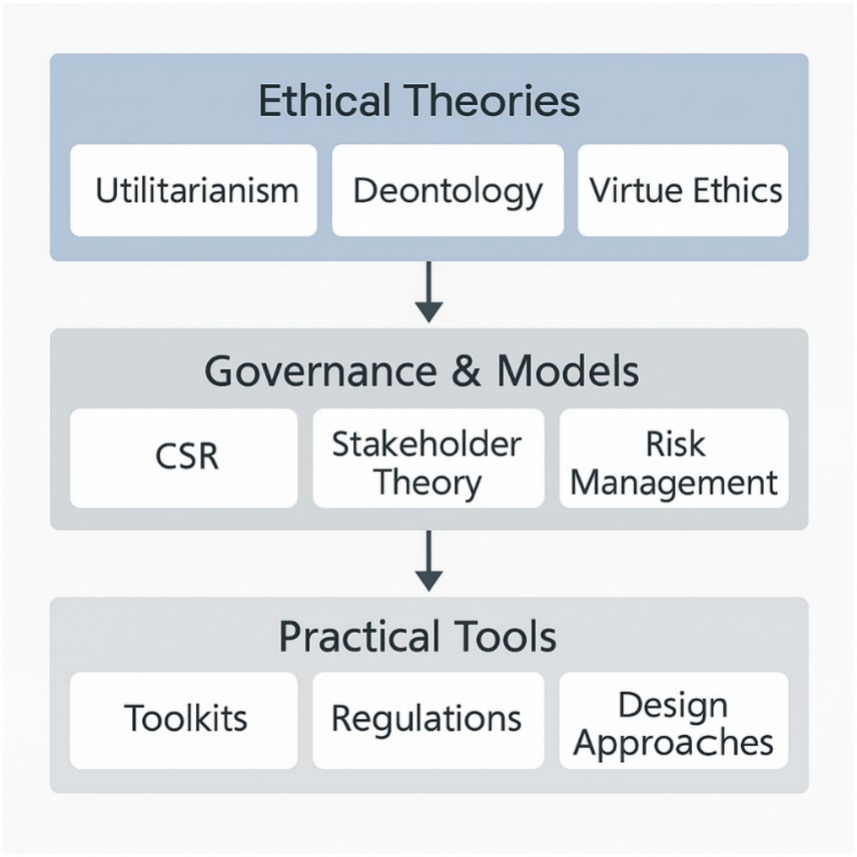
Interrelation of ethical theories, governance models, and implementation strategies.

While the previous section outlined philosophical theories underpinning responsible AI, this section shifts focus to practical models that guide implementation, including decision-making tools, CSR frameworks, and risk management systems. Figure 2 illustrates the layered relationship between ethical theories, governance frameworks, and implementation strategies discussed in this review.

### 3.1 AI ethics models

Algorithm and model ethics covers topics such as machine decision-making, algorithm selection processes, training and testing of AI models, transparency, interpretability, explainability, replicability, algorithm bias, error risk, and transparency of data flow. Predictive analytics ethics covers topics such as discriminatory decisions and contextually relevant insight. Normative ethics covers topics such as bias by generalizing AI conclusions, justice, fairness, and inequality. Relationship ethics covers topics such as user interfaces and human-computer interaction, as well as relationships between patients, doctors, and other healthcare stakeholders (Saheb et al., 2021).

There are publications that cover models, frameworks, and methodologies that AI developers can use to improve their AI ethical implementation. For example, Vakkuri et al. (2021) provide the AI maturity model for AI software. There are further publications that describe the toolbox to handle fairness in ML algorithms (Castelnovo et al., 2020) and the transparency model to develop transparent AI systems (Felzmann et al., 2020).

Pant et al. (2024) grounded theory literature evaluation provides an understanding of practitioners’ viewpoints on AI ethics. This study shows that there is not a single, widely agreed-upon concept of AI ethics, which makes practical application difficult. The results imply that in order to guarantee that AI products function ethically and within society standards, ethical issues must be incorporated into the development stages of the products. Five categories, awareness, perception, need, difficulty, and approach, are identified in the research in relation to practitioners’ experiences with ethics in AI.

Among the various applied ethics models, decision-making frameworks occupy a central role in guiding how ethical judgments are made during AI development. The next subsections analyze multiple ethical decision-making models and outline how such models support dynamic risk governance throughout the AI lifecycle.

#### 3.1.1 Ethical decision-making models

When one considers the qualitatively diverse forms that models frequently acquire, it becomes clear how difficult it is to evaluate models of ethical decision making. In specifically, the literature is dominated by three types of models. According to Kleindorfer et al. (1993), a normative model of ethical decision making places emphasis on how decision makers should ideally carry out the various steps in the decision-making process. Descriptive models of ethical decision making, on the other hand, take into account empirical data on the actual procedures that decision makers use to reach their decisions. Given the complicated context in which judgments are made, prescriptive models of ethical decision making take empirical evidence into account in an effort to assist decision makers in improving their performance (White et al., 2023).

Many models of ethical decision-making are presented in the literature, and each one provides a different perspective on the mechanisms that underlie moral judgments. For example, a thorough analysis revealed nine standard practices that were advised in 52 distinct models, indicating agreement on crucial phases in moral decision-making (Suarez et al., 2023). These procedures entail determining the ethical dilemma, obtaining pertinent data, weighing the available options, and assessing the possible outcomes of each decision. These organized methods help people make better decisions by promoting a clearer knowledge of the ethical environment. When different ethical decision-making models are compared, their methods show both commonalities and variances. For instance, although many models support a sequential approach to decision-making, others include feedback loops that enable an iterative process of reevaluating options (Cottone and Claus, 2000). Table 2 presents a comparative analysis of multiple ethical decision-making models.

**Table 2:** Comparative analysis of prominent ethical decision-making models in AI.

Authors	Model/concept	Key components	Strengths	Weaknesses/criticisms
Jones (1991)	Issue-Contingent Model	Effect concentration, proximity, extent of consequences, probability of effect, social consensus, temporal immediacy	Comprehensive Supported by research	Descriptive, not prescriptive Lacks focus on judgment
Bartlett (2003)	Ethical Model Review	Ecological validity Balance between individual/social context Focus on real-life decision processes	Real-world application Balance between context/cognition	Descriptive approach Lacks prescriptive guidance
Kelley and Elm (2003)	Critique of Jones’ Model	Organizational factors Focus on moral intensity and context	Highlights organizational role Context-driven insights	Descriptive Limited prescriptive focus
Cottone and Claus (2000)	Integrated Ethical Decision-Making	Combines theoretical and practical approaches	Bridges theory and practice	May lack specificity in certain contexts
Chorus (2015)	Discrete Choice Analysis	Utilizes statistical models to analyze moral decision-making	Provides a quantitative approach to ethics	May oversimplify complex moral dilemmas
Rosenberg and Schwartz (2019)	Problem-Solving Approach	Emphasizes multiple behaviors and outcomes in ethical situations	Offers a structured approach to complex decisions	May add complexity; less empirical support
Board BAC (2020)	BACB Ethical Decision-Making Model	Nine-step process including identifying ethical issues and evaluating options	Comprehensive framework for behavior analysts	Complexity may hinder practical application

The wider range of models here emphasizes the need to evaluate ethical decision-making frameworks using theoretical and practical criteria. Effective models integrate individual and contextual aspects, focus on cognitive and decision processes, and highlight real-world application (ecological validity). Models need to balance comprehensiveness and usability to be practical. Descriptive models can explain decision-making, but they need to become prescriptive frameworks that help practitioners make ethical decisions. As mentioned, empirical support and simplicity in execution should also be evaluated.

#### 3.1.2 AI and CSR models

CSR models advocate key ethical values, yet often remain limited to performative commitments without enforcement mechanisms. CSR’s voluntary nature can result in selective adoption, companies may embrace transparency only when reputational gains outweigh exposure to scrutiny. Research on major e-commerce enterprises shows that human-centric techniques can boost profitability and resilience amid economic downturns like the COVID-19 pandemic (Zavyalova et al., 2023). This paradigm emphasizes the role of high-tech solutions in CSR efforts to promote economic growth and social responsibility.

The relationship between CSR and financial performance through explainable AI is another important component. Using explainable AI, a Business Research study found that while CSR initiatives may not always yield immediate financial benefits, they significantly improve long-term performance for companies that excel in sustainability (Lachuer and Jabeur, 2022). In using AI for CSR, openness and accountability are crucial, according to this study. The integration of AI with other technologies like the IoT is also changing CSR. Shkalenko and Nazarenko (2024) published a report on how organizations across geographies are using AI for sustainable development. These technologies are more likely to be used successfully in regions with strong governmental support and innovation funding, improving CSR outcomes. This research suggests that technical advances are now essential to strategic CSR initiatives to achieve sustainable goals.

Organizations may manage AI deployment’s moral challenges via ethical decision-making frameworks. Fairness and non-discrimination are essential to reducing biases caused by inaccurate data or computational procedures. Floridi et al. (2021) note that fairness rules in AI systems enhance equity and decrease biases that could discriminate against underprivileged groups. Integrating transparency and interpretability into responsible AI design helps users and stakeholders trust AI systems by explaining their decisions (Larsson and Heintz, 2020). Organizations struggle to balance transparency with the intricacies of modern AI algorithms, which are hard to interpret. Mittelstadt (2019) believes that ethical accountability requires human monitoring as firms increasingly use AI for decision-making.

### 3.2 Ethical risk assessment in AI systems

**Figure 3:**
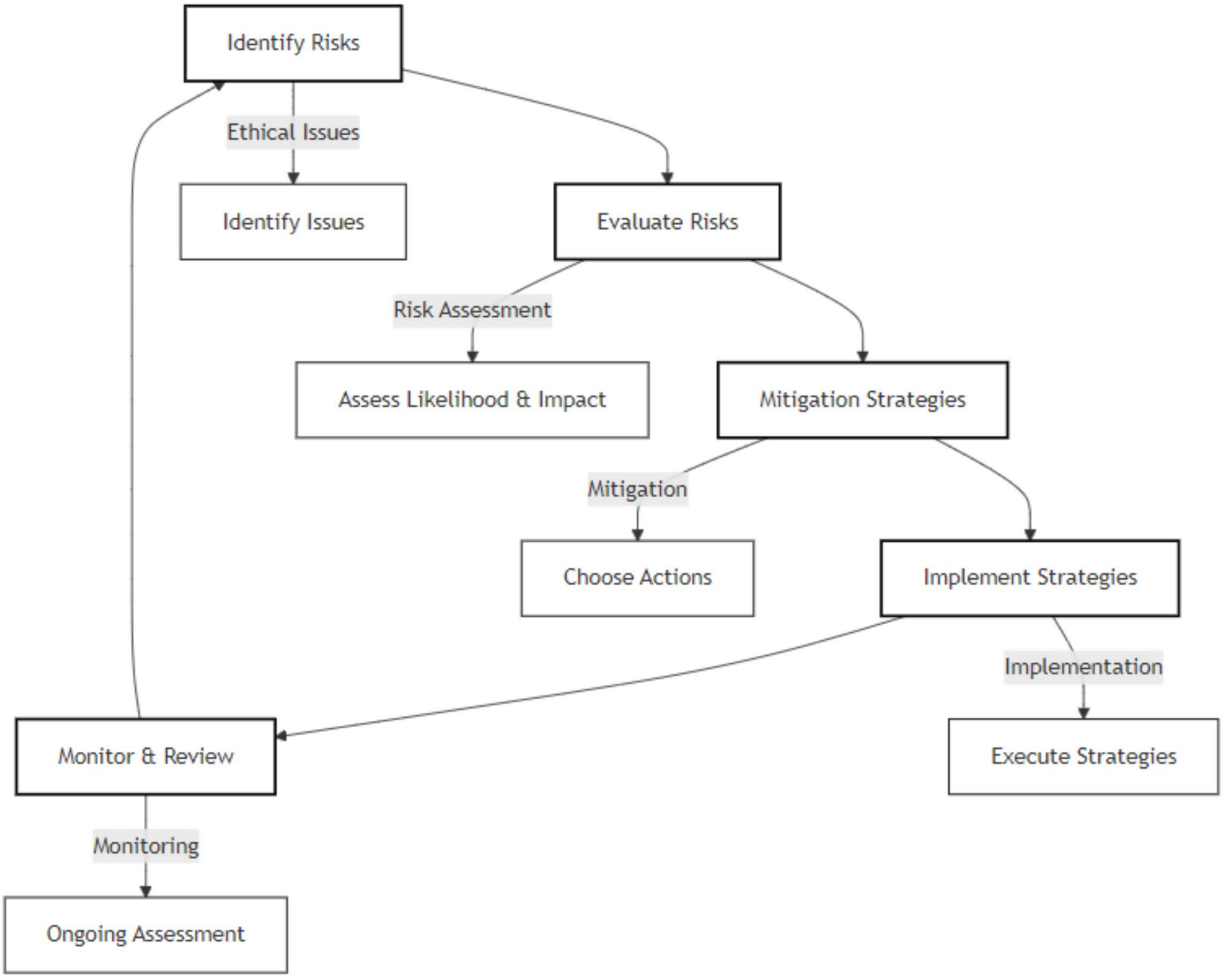
Ethical risk assessment process.

New risks arise as AI develops, necessitating flexible management techniques. An AI Risk Management Framework that highlights trustworthiness factors throughout the AI lifecycle has been proposed by the National Institute of Standards and Technology (NIST). With the potential to both create new and worsen pre-existing problems, this paradigm seeks to assist enterprises in navigating the intricacies of risk management related to generative AI technologies (AI N, 2024). The process of assessing ethical concerns in AI systems, which includes their detection, appraisal, and mitigation, is shown in Figure 3.

Although traditional enterprise risk management (ERM) frameworks offer structure, they are too static for the dynamic and unpredictable nature of AI. For example, they often fail to capture emergent risks like algorithmic discrimination or deepfake misuse, which require real-time ethical oversight and iterative updates, a need rarely addressed in current ERM implementations (Olson and Wu, 2017; Baquero et al., 2020). Their strategy consists mostly of the following steps: risk analysis (risk identification), risk assessment, risk management, and risk control. Such models operate in a very static manner, making them unsuitable for dynamic purposes such as AI (Baquero et al., 2020).

Table 3 outlines key steps in designing a risk management framework for industrial AI, including identifying and prioritizing AI use cases (Stuurman and Lachaud, 2022; Greiner et al., 2022) and evaluating existing frameworks (Butcher and Beridze, 2019). It emphasizes risk quantification (Bannister and Connolly, 2020), legal and regulatory considerations (Mäntymäki et al., 2022), and maintaining AI safety and reliability (Nikitaeva and Salem, 2022). The framework should also support innovation and enable dynamic algorithm regulation (de Almeida et al., 2021) while aligning with evolving legal standards.

**Table 3:** Key components in designing an AI risk management framework for industrial systems.

Key activity	AI use case identification & description	Prioritization of use cases	Evaluation of existing risk management frameworks	Risk measurement & quantification	Legal and regulatory considerations	Dynamic regulation of algorithms	Support for safety & reliability	Source
Use case identification	✓							Stuurman and Lachaud (2022)
Scope clarification	✓							Zhang et al. (2022)
Use of templates	✓							Brunnbauer et al. (2021)
Risk evaluation & mitigation		✓		✓				Baquero et al. (2020); Greiner et al. (2022); Lauterbach (2019)
IT system maturity assessment		✓						Mäntymäki et al. (2022)
Screening of existing frameworks			✓					Butcher and Beridze (2019)
Adapting frameworks for industry			✓					de Almeida et al. (2021); Cheatham et al. (2019); Chesterman (2019)
Risk quantification				✓				Bannister and Connolly (2020); Wirtz et al. (2020); Schneider et al. (2023)
Algorithm transparency & impact				✓				Baquero et al. (2020); Bannister and Connolly (2020)
National AI regulations					✓			Mäntymäki et al. (2022)
International AI governance rules					✓			Chambers (2021); Ellul et al. (2021)
Avoiding AI innovation restrictions					✓			Baquero et al. (2020); Wirtz et al. (2020)
Dynamic algorithm regulation						✓		Nikitaeva and Salem (2022); de Almeida et al. (2021); Chambers (2021)
Safety & reliability							✓	Nikitaeva and Salem (2022); Zhang et al. (2022)
Process flow optimization							✓	Nikitaeva and Salem (2022)
Agile AI development support							✓	Baquero et al. (2020); Wirtz et al. (2020)

### 3.3 Governance models for responsible AI

Research suggests that the development of exhaustive ethical frameworks that are specifically tailored to specific contexts can be achieved through self-regulatory efforts. Numerous organizations have established ethical guidelines that address critical issues, including algorithmic fairness, user autonomy, and data privacy (Tahaei et al., 2023). Nevertheless, critics contend that self-regulation may not possess the rigor and enforcement mechanisms required to guarantee industry-wide compliance. The absence of external oversight can lead to the inconsistent application of ethical standards and may enable companies to prioritize profit over ethical considerations (Giarmoleo et al., 2024). Advocates for government regulation contend that it is indispensable for safeguarding fundamental rights and guaranteeing accountability in AI systems, particularly those classified as “high-risk” (Khan et al., 2022).

Seven fundamental ethical precepts that AI systems must follow in order to be trusted were found through a thorough literature review: Social and Environmental Well-Being, Technical Robustness and Safety, Privacy and Data Governance, Transparency, Diversity, Non-Discrimination and Fairness, Human Agency and Oversight, and Accountability in the workplace. The High-Level Expert Group (HLEG A, 2019) on AI stated these values. The emphasis on these ideas highlights how important it is for AI systems to empower people while guaranteeing their transparent and safe operation. The difficulty is in creating objective metrics to evaluate adherence to these guidelines because subjective measurements might introduce biases and inconsistencies into ethical analyses.

Adoption of AI technologies depends heavily on trust, especially in delicate industries like finance and healthcare. A thorough analysis of the moral concerns surrounding large language models (LLMs) highlights how crucial it is to remedy inadvertent injuries, maintain openness, and adhere to human values. The report promotes a multipronged strategy that includes public involvement, industry accountability, regulatory frameworks, and ethical oversight. In order to ensure that ethical issues are integrated throughout the machine learning process, this collaborative effort is crucial for changing norms around AI development (Ferdaus et al., 2024).

There is a pressing need for standardized practices, as evidenced by the emergence of AI ethics guidelines across the globe. Significant differences in ethical principles between different jurisdictions were found in a meta-analysis of 200 governance regulations, underscoring the difficulty of creating standards that are applicable to all situations (Corrêa et al., 2023). Effective governance and accountability in AI systems may be hampered by this mismatch. A cohesive framework that may direct organizations in implementing moral practices while taking local conditions into account is called for by the review (Corrêa et al., 2023).

AI control systems are meant to monitor the actions of autonomous agents and hold them to ethical boundaries. LawZero’s approach is a good model of such a method, which seeks to build systems that can monitor and intervene in real-time when necessary. Tamang and Bora (2025) Enforcement Agent (EA) Framework is a good case in point, including supervisory agents within worlds for the purpose of monitoring and correcting other agents’ action.

The ethical consequences of autonomous systems are profound, as Amoroso and Tamburrini (2019) highlight. They touch on the necessity of human control in the operational autonomy of robot systems, particularly in high-stakes applications such as military endeavors and medicine. The challenge lies in finding a balance between the benefits of autonomy and the requirements of accountability and ethical intervention. With very advanced AI systems, the risk of misalignment with human values increases, which necessitates robust ethical structures.

### 3.4 Ethics by design approaches

Research on the design of responsible AI is essential because it deals with the nexus of technology, ethics, and society influence. The “Ethics by Design for AI” (EbD-AI) framework is a methodical way to include ethical issues into AI development. This concept places a strong emphasis on how fundamental moral principles, like liberty, privacy, justice, openness, responsibility, and well-being, should be included into AI systems at every turn of their existence. This method has been incorporated by the European Commission into its ethics evaluation processes for AI projects, emphasizing how important it is to make sure that moral values are operationalized in real-world applications rather than just being theoretical (Brey and Dainow, 2023).

A growing number of international organizations have released guidelines in response to the growing interest in AI ethics. Jobin et al. (2019) conducted a research that yielded 84 documents that delineated ethical criteria for AI. The study revealed a convergence around several core values, including responsibility, justice, transparency, and fairness. These recommendations are in keeping with the increasing consensus about the necessity of ethical frameworks that oversee AI research while also guaranteeing adherence to the law and social norms.

#### 3.4.1 Design principles for responsible AI

The proliferation of AI technology across numerous sectors has made the inclusion of ethics into AI development increasingly crucial. One popular strategy is the embedded ethics methodology, which highlights ethicists’ ongoing involvement in the AI development process. This method promotes a cooperative framework in which ethicists and developers collaborate throughout the whole development process, from planning to execution, guaranteeing that ethical issues are not just taken into account at the end but are included into every stage of the process. The goal of this proactive approach is to spot and resolve any ethical concerns as soon as possible, especially in delicate fields like healthcare where AI systems have direct contact with vulnerable populations (McLennan et al., 2022; Peterson, 2024).

Apart from the framework of embedded ethics, there exist several different methods intended to promote responsible AI. One approach being investigated is the incorporation of ethical decision-making abilities into AI systems through the use of autonomous ethical agents. These agents are made to resolve moral conundrums and choose actions that are consistent with pre-established moral standards. Nevertheless, this idea presents serious theoretical and practical difficulties for defining and putting into practice moral guidelines for machines (Peterson, 2024). Ethical standards must be contextualized within larger socio-technical frameworks, as discussed in the context of responsible AI ecosystems. According to this viewpoint, ethical concerns about the use of AI in society should not just center on specific technologies but also on their systemic effects (Stahl, 2023).

Creating accountable sociotechnical systems is another essential component of integrating ethics into AI. Empirical studies reveal that building systems that can be tested and modified is crucial to mitigating unfair risks related to the application of AI. In order to build confidence among users and stakeholders, it is necessary to ensure openness in the way AI systems function and make decisions (Pflanzer et al., 2023). Approaches that integrate ethical sensitivity into design processes have been put forth, indicating that ethical considerations can be successfully included into current risk management frameworks. Issues like bias in training data and opaque algorithmic decision-making can be lessened with this integration (Stahl, 2023).

It is recommended that organizations use a multi-layered approach to convert moral precepts into practical directives for the advancement of AI. To guarantee adherence to ethical practices, this entails creating thorough ethics codes, encouraging an ethically conscious culture within companies, and using standardization and certification procedures (Tiribelli et al., 2024). A summary of ethical frameworks and models for AI development is given in Table 4, with a focus on responsible and cooperative methods.

**Table 4:** Ethical models and frameworks for AI development.

Model	Description	Key features	Reference
Embedded ethics (EE)	A collaborative approach where ethicists and developers work together throughout the AI development process.	Continuous integration of ethical considerations Addresses ethical issues from planning to implementation	McLennan et al. (2022)
Embedded ethics for responsible AI systems (EE-RAIS)	Focuses on ethical, legal, and social values in AI systems, particularly in disaster management.	Four platforms: educational, cross-functional, developmental, algorithmic Metrics: ethical intelligence, legal intelligence, social–emotional competency, artificial wisdom	Afroogh et al. (2023)
Human-in-the-loop (HITL)	Incorporates human oversight at various stages of AI development to ensure ethical outputs.	Regular review of AI outputs Balances commercial goals with social impacts	Middleton et al. (2022)
Responsible intelligent systems	Emphasizes moral responsibility and accountability in the use of intelligent systems.	Concept of ecosystems in AI Higher-level responsibility or ‘meta-responsibility’	Stahl (2023)
Socio-technical approaches	Integrates social and technical perspectives to address ethical issues in AI development.	Collaboration across disciplines Focus on societal implications of AI technologies	McLennan et al. (2022); McLennan et al. (2024)

### 3.5 Limitations of existing responsible AI frameworks

One of the primary limitations of existing responsible AI frameworks is the lack of appropriate evaluation mechanisms. Reddy et al. (2021) highlight that AI systems in healthcare have been developed without a comprehensive evaluation of their translational aspects, such as functionality, utility, and ethics. The Translational Evaluation of Healthcare AI (TEHAI) framework was proposed to address these gaps by focusing on capability, utility, and adoption. However, the limited focus of most existing frameworks on reporting and regulatory aspects risks overlooking the day-to-day application of ethical principles in practice.

The ethical challenges associated with AI use are particularly acute in specialist areas such as palliative care. De Panfilis et al. (2023) discuss the ethical issues of AI-based clinical decision-making systems in palliative care, advocating for an equilibrium position that honors patient autonomy, quality of life, and psychosocial context of care. AI application for mortality prediction, while beneficial, is concerning due to the potential for oversimplification in complex decision-making. This mirrors a broader issue in which ethical frameworks may be unable to address the specific challenges posed by AI across a range of healthcare environments.

Doyen and Dadario (2022) outline several technical and operational pitfalls that are to blame for the low clinical impact of AI technologies. These include pitfalls related to data quality, algorithmic bias, and the failure to integrate AI systems with existing workflows. The authors argue that without addressing such underlying issues, ethical frameworks alone cannot ensure effective implementation of AI in healthcare. The need for an integrated approach incorporating both ethical and practical implementation aspects is necessary for overcoming such obstacles.

Another important consideration in the adoption of AI tools in healthcare is their trustworthiness. Lekadir et al. (2025) highlight the need to establish trust between patients, clinicians, and healthcare organizations. The FUTURE-AI guideline, which was developed by international consensus, provides best practices for developing responsible AI tools. The challenge, however, lies in translating these guidelines into effective practice since trust would typically be established with repeated and transparent interactions with AI systems.

The influence of stakeholders on the development and implementation of responsible AI frameworks is a major concern. Heymans and Heyman (2024) argue that existing guidelines have a tendency to codify the agendas of powerful stakeholders rather than the interest of the broader public. This can lead to ethical frameworks that are disconnected from practical situations, undermining their usefulness. A more extensive approach that involves diverse stakeholder perspectives is needed to create ethical guidelines that are grounded in the realities of AI adoption.

Medical education is another area where ethical frameworks are confronted with the introduction of AI. Ma et al. (2024) stress overall AI literacy among medical students as a precursor to secure and responsible AI-assisted patient care. Education today tends to focus more on the ethical aspects than the technical proficiency, and as a result, there exists a knowledge gap in assessing and deploying AI technologies in clinical practice. Such educational gaps are essential to be addressed to develop a workforce capable of handling the intricacies of AI in healthcare.

## 4 Implementation strategies for responsible AI adoption

The adoption of responsible AI in industry is critical, as proven by several case studies highlighting both triumphs and failures. For example, Meta’s collaboration with researchers to create responsible AI seminars offers a proactive strategy to teaching practitioners on ethical standards, boosting engagement and motivation to adopt responsible AI principles in their work (Stoyanovich et al., 2024). Significant ethical failures, such as the Uber autonomous vehicle issue, highlight the critical need for strong ethical frameworks to reduce risks such as bias and privacy violations (Firmansyah et al., 2024). Practitioners frequently struggle to use these frameworks effectively, revealing a deficit in tools and knowledge (Baldassarre et al., 2024). This highlights the significance of responsible AI in modern business dynamics. Including ethical considerations not only improves operational efficiency but also acts as a buffer against potential ethical problems (Tariq MU, 2024).

To ensure responsible AI deployment across multiple sectors, best practice guidelines and toolkits for responsible AI development are essential. In order to improve fairness and transparency in AI systems, these toolkits should incorporate Value Sensitive Design (VSD) concepts, which place an emphasis on human values throughout the technological design process (Sadek et al., 2024). International standards, like those from IEEE and ISO, are essential for standardizing moral behavior, encouraging cooperation between interested parties, and guaranteeing that AI applications respect human rights and society norms (Firmansyah et al., 2024). Diverse viewpoints can reduce biases and improve the ethical governance of AI systems when they are included into AI development (Zhao et al., 2023).

The adoption of responsible AI is significantly influenced by organizational culture, and leadership is a key component in creating an atmosphere where moral principles are given priority. A culture of ethics is shaped within a company by ethical leaders who act as role models, impacting the conduct and output of their subordinates (Muktamar, 2023). They are in charge of creating plans that encourage moral conduct and putting standards of ethics into effect that direct AI procedures (Muktamar, 2023). Effective training and development initiatives that place a strong emphasis on moral reasoning and character development for staff members are also crucial for fostering responsible AI activities (Kuennen, 2023). The creation of responsible AI can be aided by a strong corporate culture that places a great emphasis on perceived justice. This can result in more ethical outcomes when using AI (Mohammadabbasi et al., 2022).

As businesses work through the complexity of AI regulations, the confluence between responsible AI and compliance with data protection rules becomes more and more important. Global norms for responsible AI use are inspired by the EU AI Act, which provides a groundbreaking framework for risk-based AI governance that prioritizes ethical considerations and adherence to data protection rules (Eu, 2024; Matai, 2024). This act highlights the necessity for expert collaboration to handle compliance difficulties, outlining requirements for AI developers and requiring interdisciplinary governance to successfully execute its rules (Zhong, 2024). The body of research highlights the significance of a human-centric approach to responsible AI, emphasizing ethics, privacy, and security to make sure that AI systems are created and implemented in a way that upholds social norms and individual rights (Goellner et al., 2024).

A case study is the utilization of AI in Alibaba’s intelligent warehouse, where efficient orchestration of resources results in successful AI implementation. The study emphatically states controlling AI technology, individuals, and processes to create value. Key AI assets such as data and algorithms were orchestrated with existing systems effectively, resulting in enhanced operational effectiveness and efficiency (Zhang et al., 2021). Successful example is the use of AI in the context of CSR practices of Spanish companies. Internet of Things (IoT) has been employed to enhance the implementation of CSR strategies, demonstrating technology usage to make business more ethical. Empirical tools developed in this study provide insights on how firms can utilize AI to enable them to achieve their CSR objectives effectively (Mattera, 2020).

Conversely, there are a number of instances where AI ethics implementations failed due to the lack of adequate knowledge on the ethical impacts of AI technologies. For example, in research on the ethical and regulatory concerns of AI technologies in healthcare, it was revealed that most implementations did not adequately protect individual health data. The research indicated that algorithmic or data management practices created errors that led to severe ethical violations, and thus there is a need for good ethical guidelines when implementing AI (Mennella et al., 2024). Research into the bias of AI algorithms has indicated that ethical deployments have been a failure. A study indicated that AI system discrimination is often a consequence of limited data sets as well as developers’ backgrounds, leading to biased results. This highlights the need for diverse data and inclusive design practices in order to mitigate bias and ensure fairness in AI systems (Chen, 2023).

The two contradictory implications of these case studies provide rich lessons to organizations looking to implement AI responsibly. Successful implementations all share some characteristic commonalities, including a clear correspondence of AI programs to organizational values and commitment to transparency and accountability. For instance, the Liverpool Football Club example demonstrates how human expertise and data analytics might come together to help bring about a sustained competitive advantage, and in doing so emphasize the importance of human-AI collaboration toward achieving ethical solutions (Lichtenthaler, 2020).

On the other hand, failed implementation usually reveals inherent deficiencies in the understanding of AI technologies’ ethical aspects. Ethical values should be given the prime consideration at the outset, with fairness, transparency, and accountability being built into AI systems. Lessons learned from these case studies emphasize the necessity to develop end-to-end ethical approaches that guide AI deployment across various sectors.

## 5 Conclusion

As enterprises and communities navigate the rapid growth of AI, integrating ethics into AI adoption is not only a normative imperative but also a strategic enabler for organizational success. This review confirms that ethical AI adoption, anchored in established theories such as utilitarianism, deontology, and virtue ethics, provides a philosophical foundation for assessing responsibility, fairness, and transparency in practice. In alignment with the first objective, we demonstrated how ethical theories underpin responsible AI by examining their operationalization through governance models and stakeholder responsibility frameworks. These models provide normative direction for AI implementation, addressing risk, bias, and decision accountability in alignment with research from Jobin et al. (2019), Hagendorff (2020), and Khan et al. (2022). Regarding the second and third objectives, evaluating governance models and decision-making tools, we identified multilayered frameworks such as Ethics by Design (EbD-AI) and Embedded Ethics, and structured models for ethical risk assessment that support both ethical compliance and strategic innovation. These frameworks, as discussed in works by Brey and Dainow (2023) and the NIST AI N (2024), demonstrate the synergy between ethical conduct and scalable, responsible adoption. In response to the final objective, the article provides a strategic perspective on implementation approaches by drawing on real-world cases, e.g., Alibaba’s intelligent warehouse (Zhang et al., 2021), showing that ethics-infused strategy can drive sustainable performance and trust-based innovation. These findings reinforce the connection between ethical AI practices and organizational success, a concept originally emphasized in the article’s title. Therefore, the study affirms that successful AI adoption depends not only on technical excellence but on integrated ethical strategies that align with stakeholder expectations, governance mechanisms, and evolving regulatory standards. Bridging theory and practice through such integration is essential for building trustworthy AI systems that support both innovation and the long-term success of organizations.
